# Supplementary material for: PLSCR1 is a cell-autonomous defence factor against SARS-CoV-2 infection
Source: Nature. 2023 Jul 12;619(7971):819–27. doi: 10.1038/s41586-023-06322-y (PMC10371867; doi:10.1038/s41586-023-06322-y)
Supplement: Supplementary file 1 — This file contains Supplementary Fig. 1: Gel source data; and Supplementary Fig. 2: Flow cytometric gating strategy. [file 41586_2023_6322_MOESM1_ESM.pdf]

nature portfolio

https://doi.org/10.1038/s41586-023-06322-y

Supplementary information

PLSCR1 is a cell-autonomous defence factor against SARS-CoV-2 infection

In the format provided by the authors and unedited

Supplementary Fig. 1 Source data for Western blots

Fig. 3e

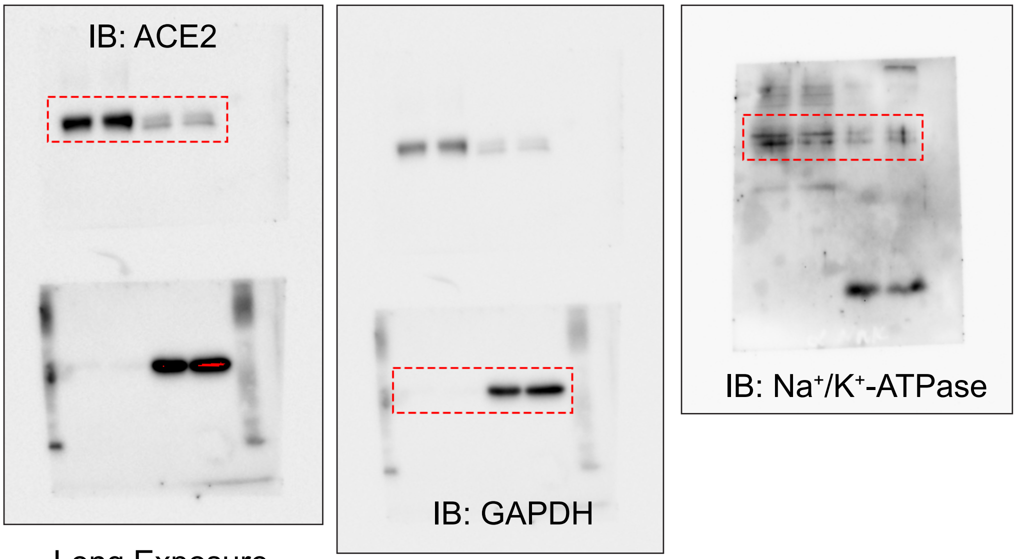

Long Exposure

Short Exposure

Fig. 3g

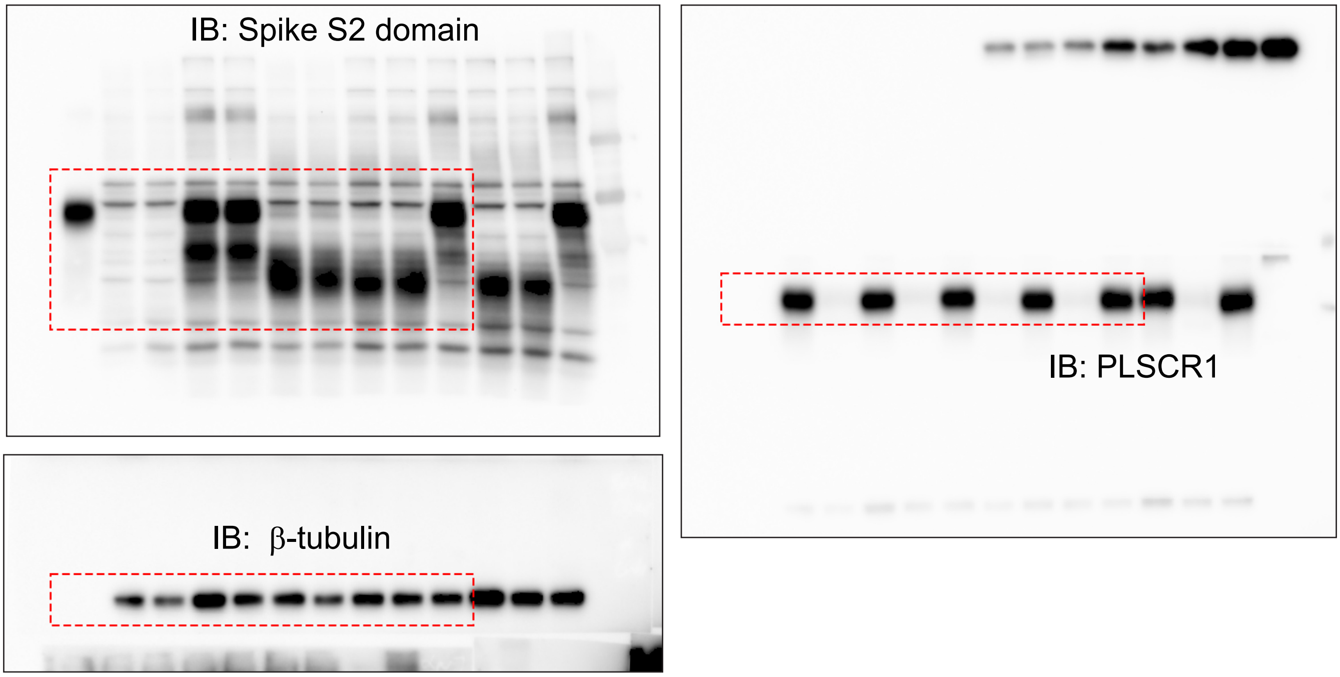

Fig. 4d

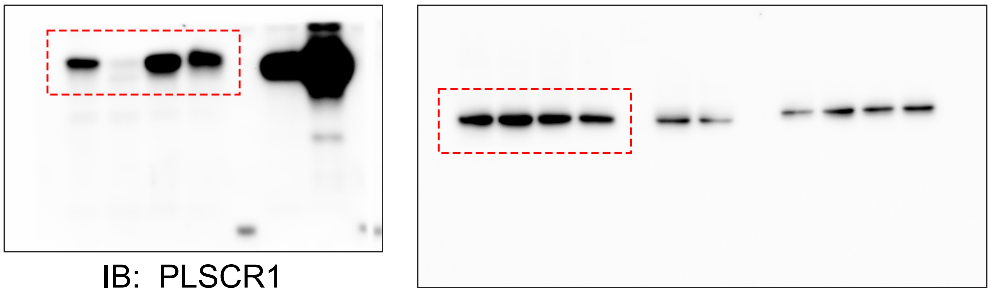

IB: PLSCR1

IB: β-tubulin

Extended Data Fig.1

ED Fig. 1d

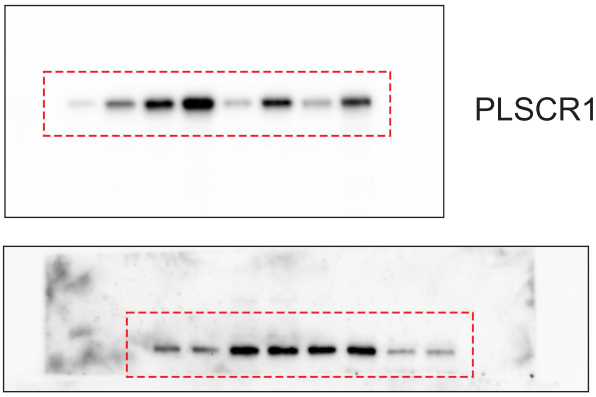

PLSCR1

GAPDH

ED Fig. 1e

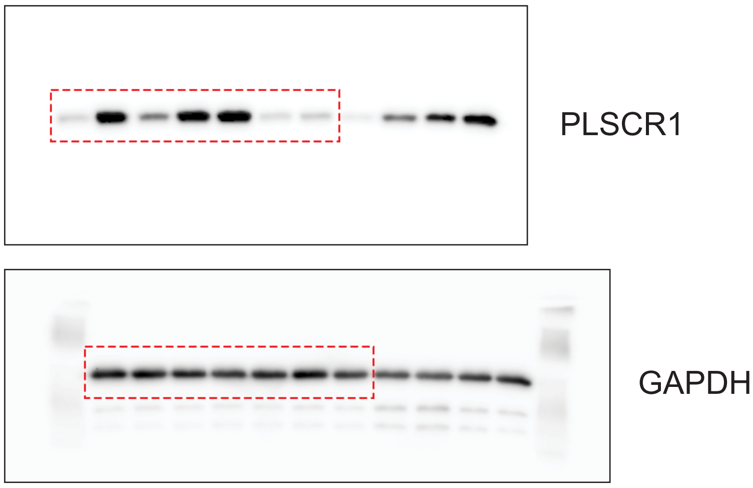

PLSCR1

GAPDH

Extended Data Fig.2

ED Fig. 2a

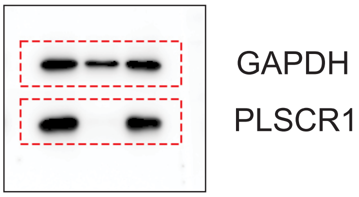

ED Fig. 2b

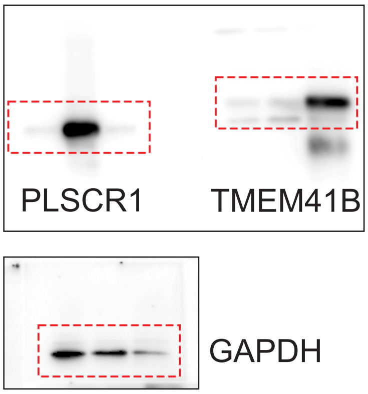

ED Fig. 2c

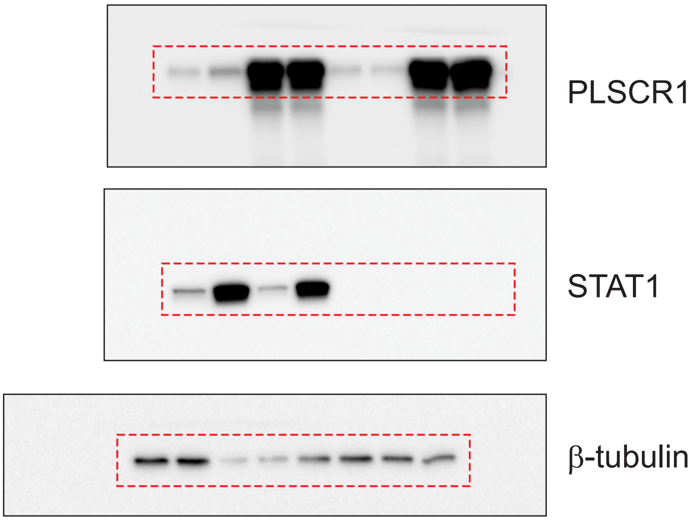

ED Fig. 2e

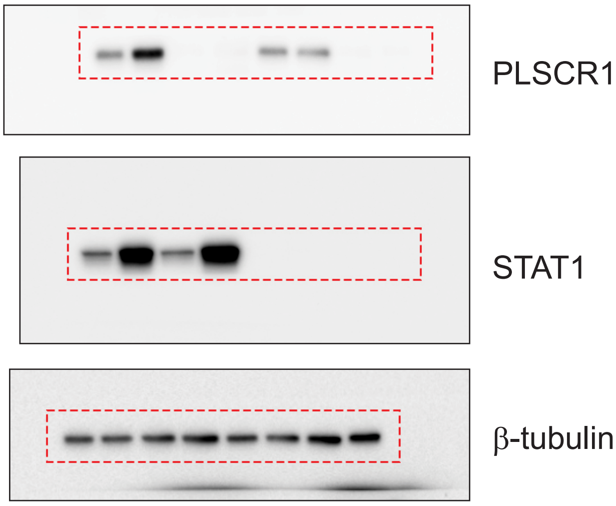

ED Fig. 2f

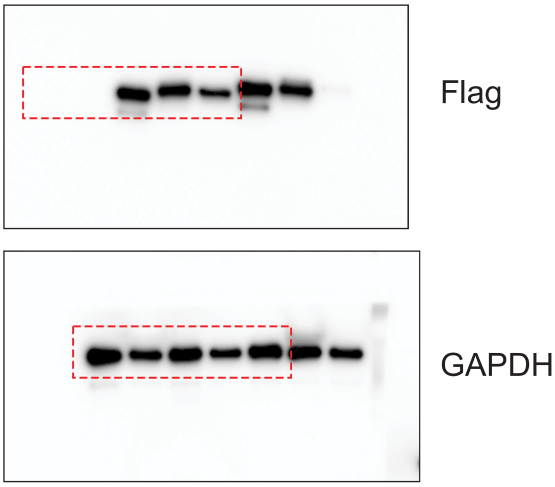

ED Fig. 2g

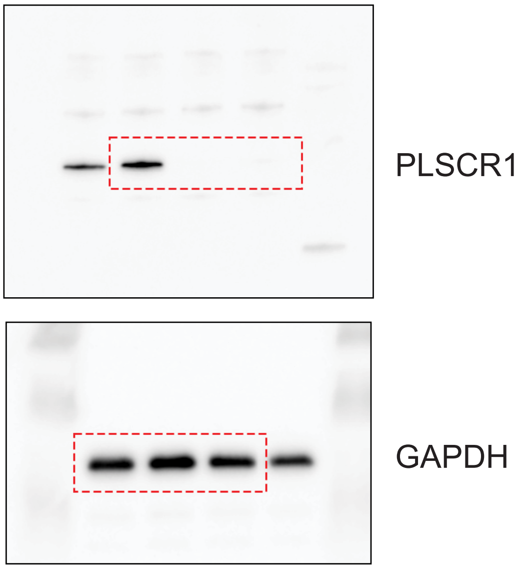

Extended Data Fig.3

ED. Fig.3b

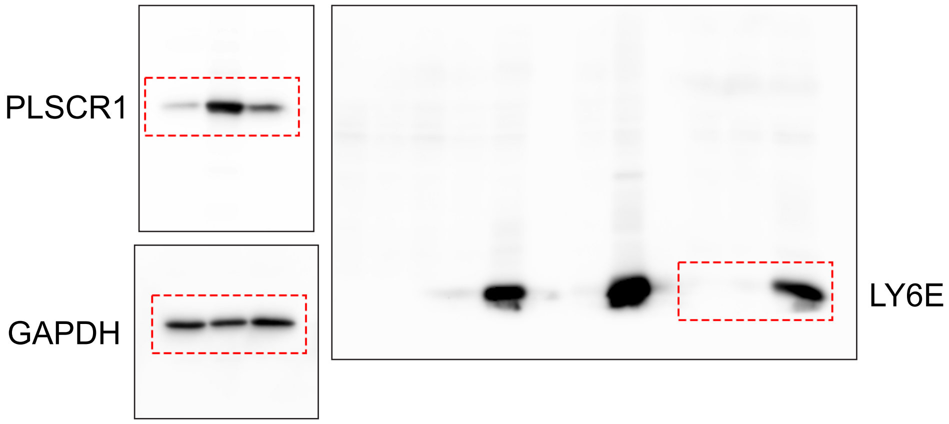

ED. Fig.3c

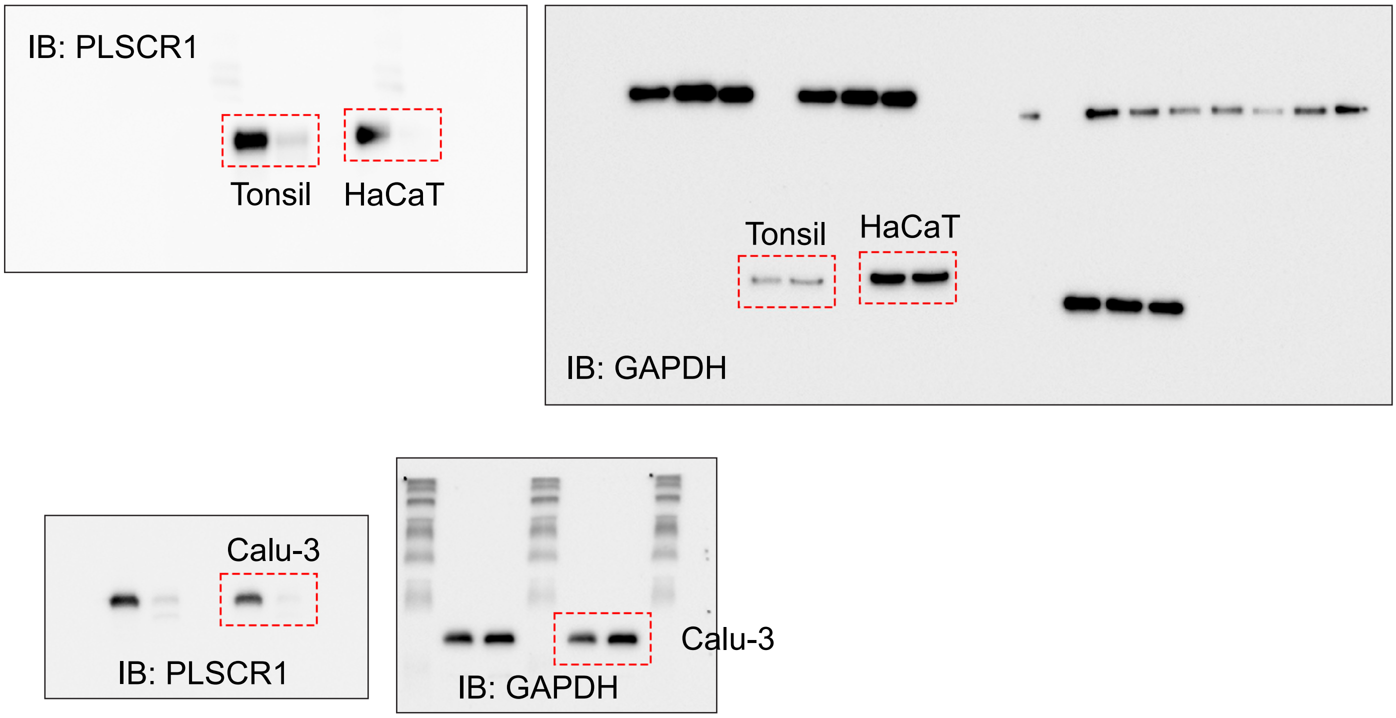

ED. Fig.3d

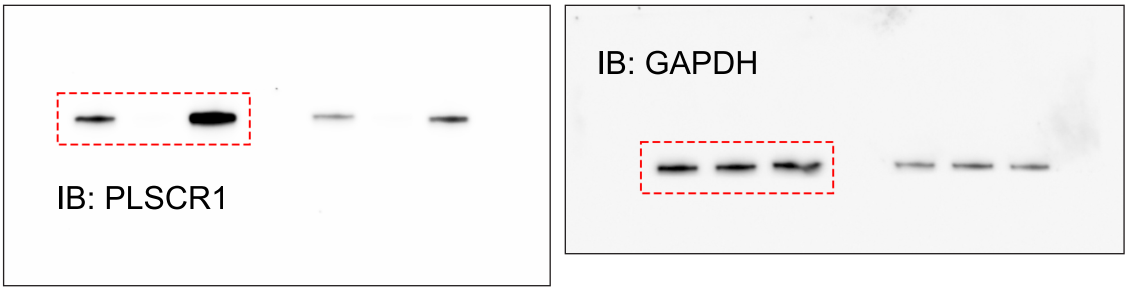

Extended Data Fig.3

ED. Fig.3f

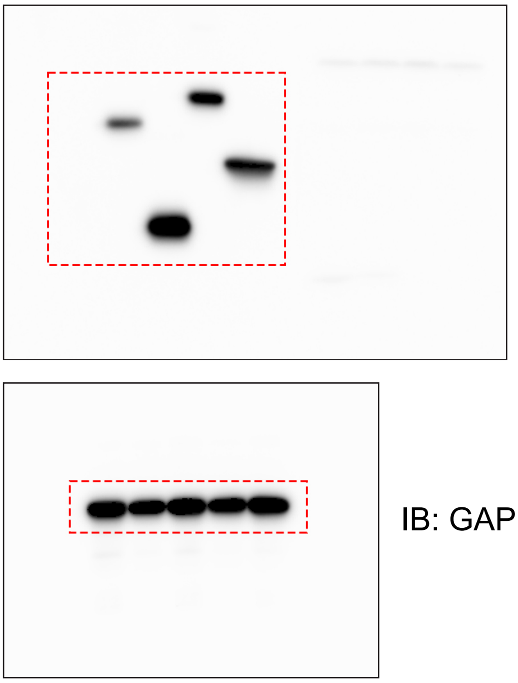

IB: Flag

IB: GAPDH

ED. Fig.3g

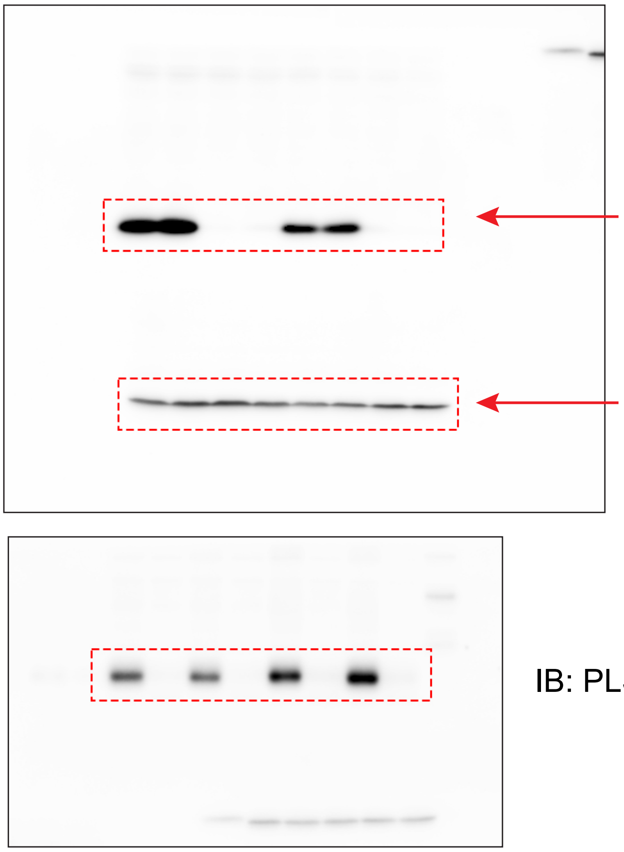

IB: LY6E

IB: β-tubulin

IB: PLSCR1

Extended Data Fig. 4

ED. Fig. 4d

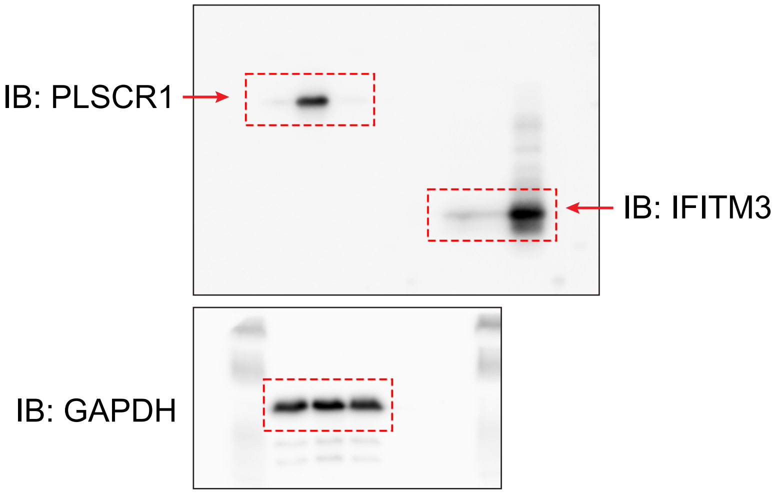

Extended Data Fig. 5

ED. Fig. 5d

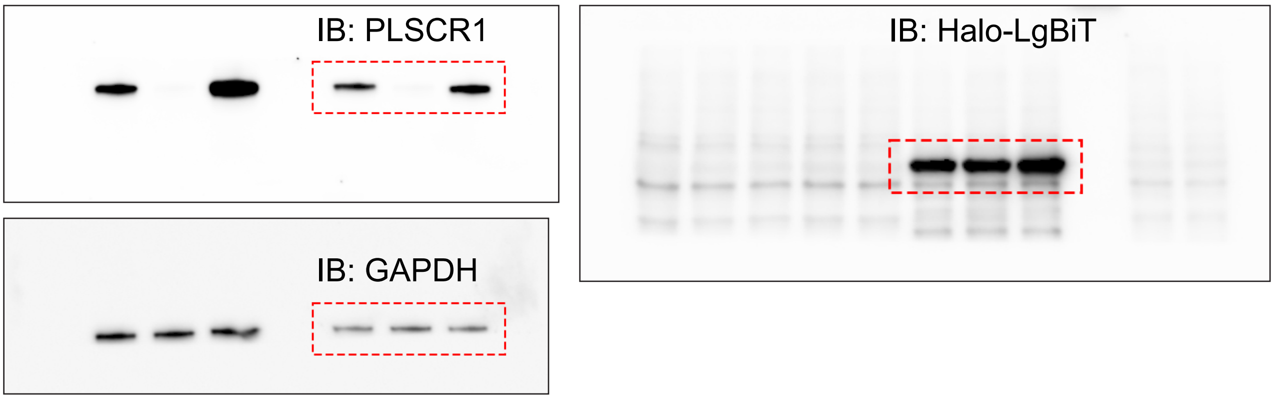

Extended Data Fig.8

ED. Fig. 8d

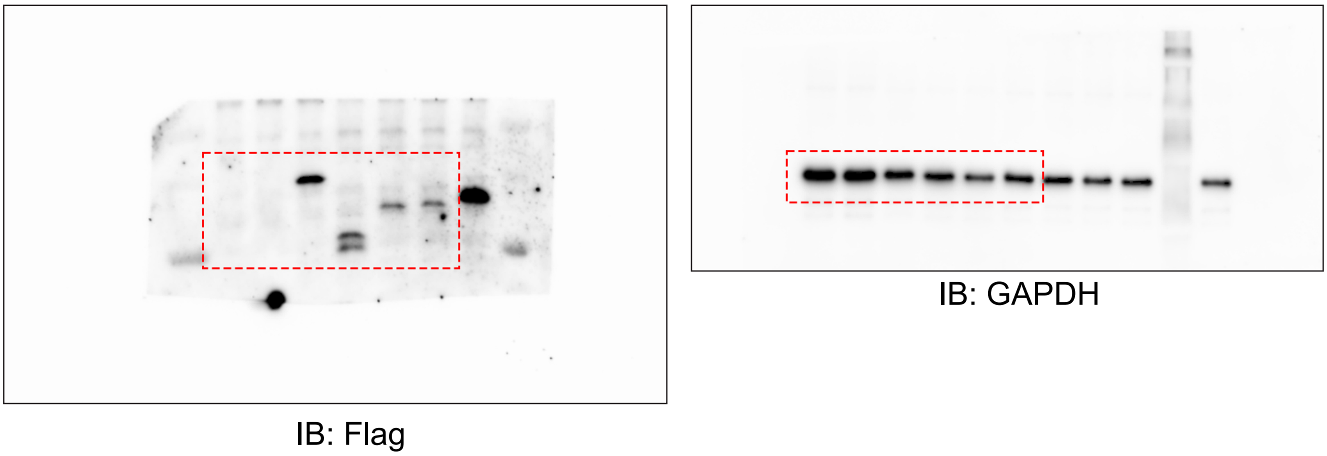

ED. Fig. 8e

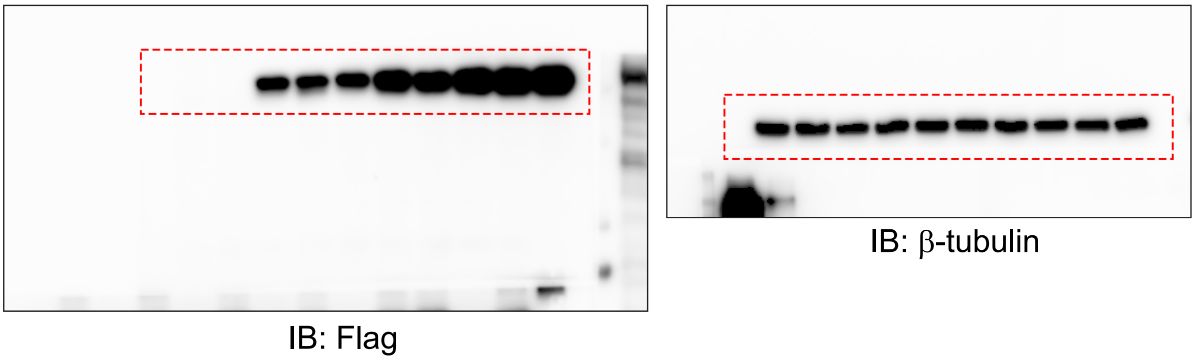

ED. Fig. 8f

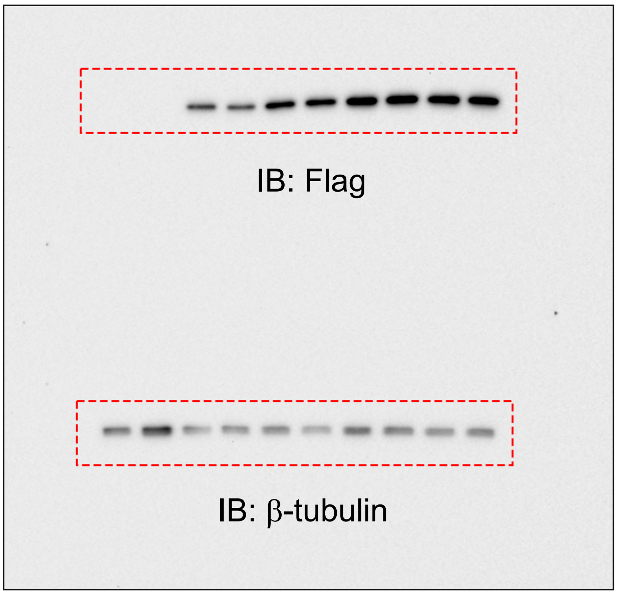

ED. Fig.8h

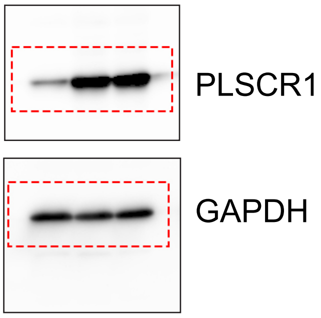

Extended Data Fig. 9

ED Fig. 9a

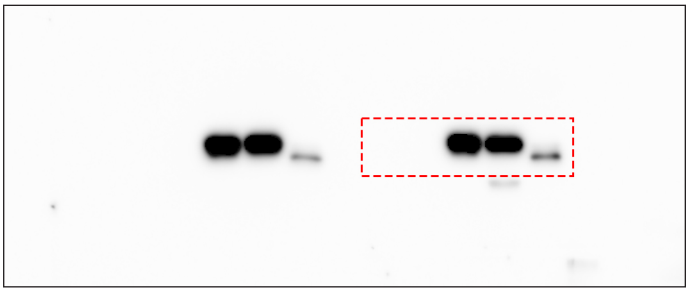

IB: Flag

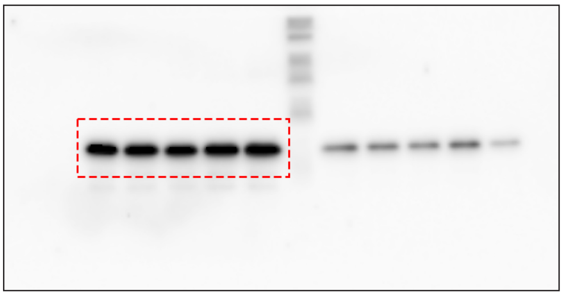

IB: GAPDH

ED Fig. 9d

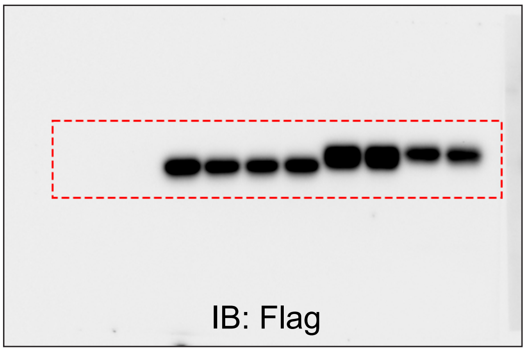

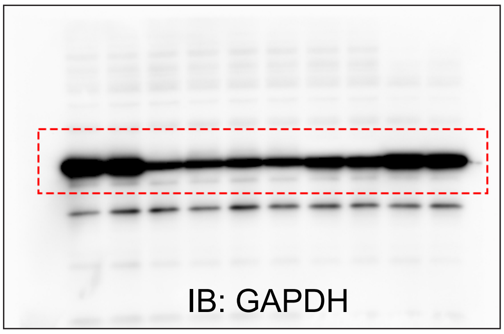

ED Fig. 9e

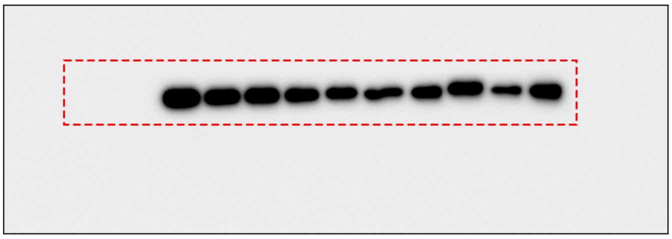

IB: Flag

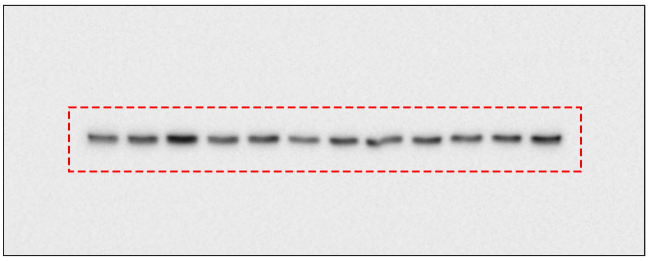

IB: β-tubulin

Extended Data Fig. 10

ED Fig. 10d

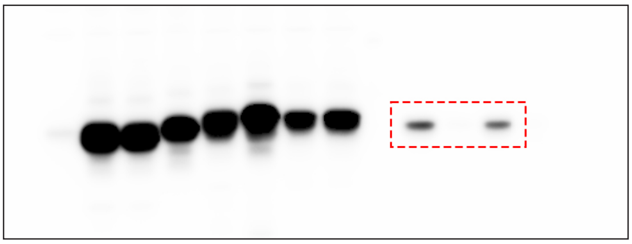

IB: PLSCR1

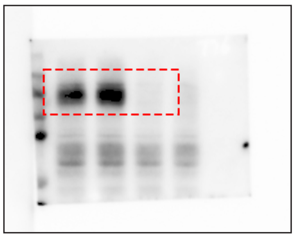

IB: TMEM16F

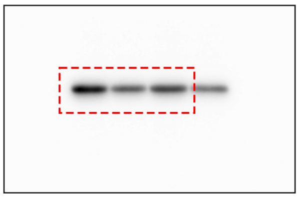

IB: GAPDH

ED Fig. 10e

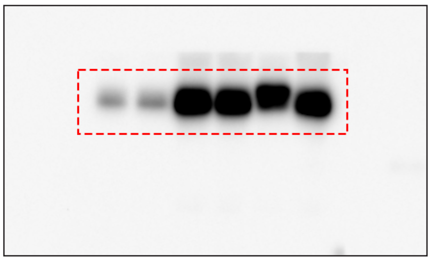

IB: PLSCR1

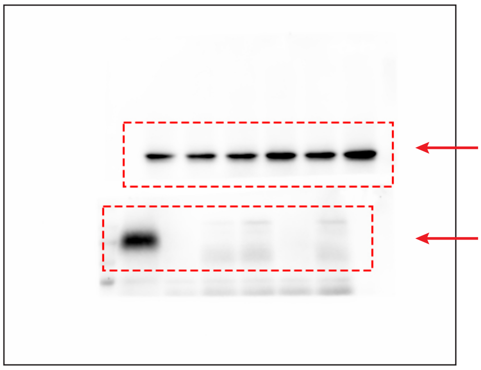

IB: β-tubulin

IB: TMEM16F

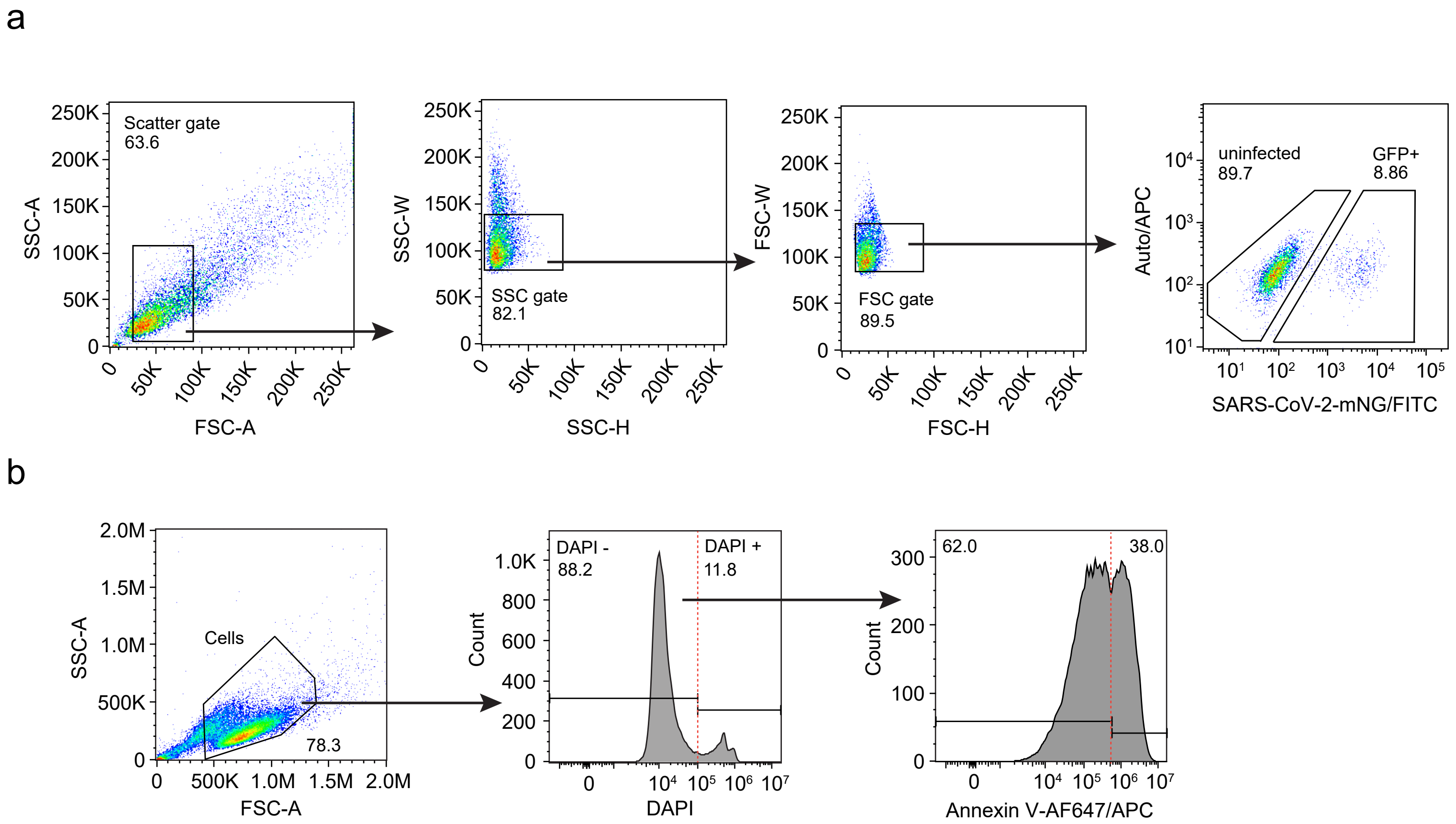

Supplementary Figure 2. Flow cytometric gating and sorting strategies.
a, Flow cytometric sorting strategy for genome-wide CRISPR screening in Huh7.5 and A549-ACE2 cells. Related to **Fig.1d**. **b**, Flow cytometric gating strategy for PS externalization analysis. Related to **Fig. 4g,h**.
