# Supplementary material for: PLSCR1 is a cell-autonomous defence factor against SARS-CoV-2 infection
Source: Nature. 2023 Jul 12;619(7971):819–27. doi: 10.1038/s41586-023-06322-y (PMC10371867; doi:10.1038/s41586-023-06322-y)
Supplement: Supplementary file 2 — Reporting Summary [file 41586_2023_6322_MOESM2_ESM.pdf]

nature portfolio

Corresponding author(s): John D. MacMicking

Last updated by author(s): May 22, 2023

Reporting Summary

Nature Portfolio wishes to improve the reproducibility of the work that we publish. This form provides structure for consistency and transparency in reporting. For further information on Nature Portfolio policies, see our Editorial Policies and the Editorial Policy Checklist.

Statistics

For all statistical analyses, confirm that the following items are present in the figure legend, table legend, main text, or Methods section.

n/a	Confirmed	
☐	☒	The exact sample size (*n*) for each experimental group/condition, given as a discrete number and unit of measurement
☐	☒	A statement on whether measurements were taken from distinct samples or whether the same sample was measured repeatedly
☐	☒	The statistical test(s) used AND whether they are one- or two-sided<br>*Only common tests should be described solely by name; describe more complex techniques in the Methods section.*
☒	☐	A description of all covariates tested
☐	☒	A description of any assumptions or corrections, such as tests of normality and adjustment for multiple comparisons
☐	☒	A full description of the statistical parameters including central tendency (e.g. means) or other basic estimates (e.g. regression coefficient) AND variation (e.g. standard deviation) or associated estimates of uncertainty (e.g. confidence intervals)
☐	☒	For null hypothesis testing, the test statistic (e.g. F, t, r) with confidence intervals, effect sizes, degrees of freedom and P value noted<br>*Give P values as exact values whenever suitable.*
☒	☐	For Bayesian analysis, information on the choice of priors and Markov chain Monte Carlo settings
☒	☐	For hierarchical and complex designs, identification of the appropriate level for tests and full reporting of outcomes
☒	☐	Estimates of effect sizes (e.g. Cohen's d, Pearson's r), indicating how they were calculated

Our web collection on statistics for biologists contains articles on many of the points above.

Software and code

Policy information about availability of computer code

Data collection	SoftMax® Pro Software (v.7) was used to collect data from microplate reader. BD FACSDiva (v.8) was used to collect flow cytometry data. Bio-Rad Image Lab Touch Software (v.2.2.0.08) was used to collect images of protein and DNA gels. Microscopy images were obtained using LAS X (v 3.7.4). Gen5 (BioTek) and CellReporterXpress (Molecular devices) were used to collect fluorescence images of 96-well plates.
Data analysis	GraphPad Prism 9 was used to generate all graphs and perform statistical analysis.<br>LAS X (v 3.7.4), FUJI/ImageJ or Imaris 9.8 were used to process confocal images.<br>Gen5 (BioTek) and CellReporterXpress (Molecular devices) were used to process high-content images of 96-well plates.<br>FlowJo 10.8.1 was used to process and visualize FACS data.<br>SoftWoRx (v7.0) was used to process live cell images.<br>Vutara SRX 7.0.06 software was used to process 4Pi single-molecule switching (4Pi-SMS) images.<br>Whole-genome CRISPR/Cas9 screening data was analyzed using MAGeCK (v.0.5.6).<br>RNA seq was analyzed using STAR (version 2.8), DESeq2 (version 3.9) and R version 3.5.0.<br>Microsoft Excel 2010 was used to record and store data.<br>ChimeraX 1.4 (https://www.rbvi.ucsf.edu/chimerax/) was used to analyze protein structures.<br>GROMACS version 2021.3 was used for molecular dynamic simulation and RMSF analysis.<br>Coarse-grained simulation was analyzed using Memembed 1.15, Insane 3, Martinize 2 and Python version 3.7.<br>PyMOL (2.3.0) was used for RMSF and H-bond visualization<br>VMD (1.9.4) was used for the hydrogen bond analysis and movie creation<br>The membrane bending rigidity data was analyzed using MATLAB 9.13 and Origin 2023.

For manuscripts utilizing custom algorithms or software that are central to the research but not yet described in published literature, software must be made available to editors and reviewers. We strongly encourage code deposition in a community repository (e.g. GitHub). See the Nature Portfolio guidelines for submitting code & software for further information.

Data

Policy information about availability of data

All manuscripts must include a data availability statement. This statement should provide the following information, where applicable:

- Accession codes, unique identifiers, or web links for publicly available datasets
- A description of any restrictions on data availability
- For clinical datasets or third party data, please ensure that the statement adheres to our policy

The data supporting the findings of this study are available within the paper and its Supplementary Information files. Genome-wide CRISPR screening data are provided in Supplementary Tables 2 and 3. RNA-seq data are provided in Supplementary Tables 4 and 5. Full versions of all blots are provided in Supplementary Fig. 1. The gating strategies of flow cytometry are provided in Supplementary Fig. 2. The human genome reference (hg38) used in RNA-seq analysis is available in the NCBI genome assembly with accession number GCF_000001405.39. The protein expression profiles are available in the web-based Human Protein Atlas database (https://www.proteinatlas.org/). The transcriptional factor binding profiles are available in the web-based JASPAR database (https://jaspar.genereg.net/). The Materials, reagents or other experimental data are available from the corresponding author upon request. Source data are provided with this paper.

Human research participants

Policy information about studies involving human research participants and Sex and Gender in Research.

Reporting on sex and gender	N/A
Population characteristics	N/A
Recruitment	N/A
Ethics oversight	N/A

Note that full information on the approval of the study protocol must also be provided in the manuscript.

Field-specific reporting

Please select the one below that is the best fit for your research. If you are not sure, read the appropriate sections before making your selection.

☒ Life sciences ☐ Behavioural & social sciences ☐ Ecological, evolutionary & environmental sciences

For a reference copy of the document with all sections, see nature.com/documents/nr-reporting-summary-flat.pdf

Life sciences study design

All studies must disclose on these points even when the disclosure is negative.

Sample size	The sample size and the results of statistical analyses are described in the relevant figure legends. Sample size was determined based on experimental trials and with consideration of previous publications on similar experiments (ref. 22, 23, 24, 25) to allow for confident statistical analyses. No statistical methods were used to predetermine sample sizes.
Data exclusions	No data was excluded.
Replication	All experimental findings were replicated at least three times unless otherwise mentioned in figure legends.
Randomization	For validation of whole-genome screening candidates and the experiments measuring the function of PLSCR1 mutants, cells of each genotype were seeded on culture plate in random orders or randomly assigned to different treatments. Otherwise, randomization was not performed. Data variability was controlled by multiple biological replicates and multiple technical replicates within an experiment. Cells were grown under the same conditions in dishes or plates to minimize unexpected environmental variations.
Blinding	For validation of whole-genome screening candidates, samples were labeled as code and were blind to the individual who performed the experiment. For microscopy data collection, the samples of different treatments or genotypes were randomly selected for imaging. The fields of view were chosen on a random basis. For other experiments, blinding was not performed because different genotypes or wells require different treatments or conditions.

Reporting for specific materials, systems and methods

We require information from authors about some types of materials, experimental systems and methods used in many studies. Here, indicate whether each material, system or method listed is relevant to your study. If you are not sure if a list item applies to your research, read the appropriate section before selecting a response.

Materials & experimental systems

n/a	Involved in the study
☐	☒ Antibodies
☐	☒ Eukaryotic cell lines
☒	☐ Palaeontology and archaeology
☒	☐ Animals and other organisms
☒	☐ Clinical data
☒	☐ Dual use research of concern

Methods

n/a	Involved in the study
☒	☐ ChIP-seq
☐	☒ Flow cytometry
☒	☐ MRI-based neuroimaging

Antibodies

Antibodies used

The following antibodies were purchased from Proteintech:
Rabbit anti-GAPDH monoclonal antibody (Clone 1E6D9) (60004-1-Ig) (WB: 1:2000)
Mouse anti-GFP tag monoclonal antibody (Clone 1E10H7) (66002-1-Ig) (WB: 1:2000)
Rabbit anti-PLSCR1 polyclonal antibody (11582-1-AP) (WB: 1:2000)
Mouse anti-Halo tag monoclonal antibody (Clone 28a8) (WB: 1:500)
Rabbit anti-TMEM41B polyclonal antibody (29270-1-AP) (WB: 1:2000)
Rabbit anti-IFITM3 polyclonal antibody (11714-1-AP) (WB: 1:2000)

The following antibodies were purchased from Cell Signaling:
Rabbit anti-Na,K-ATPase polyclonal antibody (#3010S) (WB:1:1000)
Rabbit anti-Flag tag monoclonal antibody (Clone D6W58) (14793S) (WB: 1:2000)
Rabbit anti-β-Tubulin monoclonal antibody (Clone 9F3) (#2128S) (WB: 1:1000)

The following antibody was purchased from R&D Systems:
Goat anti-ACE2 polyclonal antibody (AF933) (WB: 1:4000)

The following antibodies were purchased from Sigma-Aldrich:
Mouse anti-PLSCR1 monoclonal antibody (Clone 4D2) (MABS483) (IFA: 1:200)
Mouse anti-dsRNA monoclonal antibody (Clone rJ2) (MABE1134) (IFA: 1:200)
Rabbit anti-TMEM16F polyclonal antibody (HPA038958) (WB: 1:2000)
Sheep anti-mouse IgG horse-radish peroxidase-conjugated secondary antibody (GENXA931-1ML) (WB:1:5000)
Sheep anti-rabbit IgG horse-radish peroxidase-conjugated secondary antibody (GENA934-1ML) (WB:1:5000)

The following antibody was purchased from Sino Biological:
Rabbit anti-SARS-CoV-2 nucleocapsid monoclonal antibody (Clone 019) (40143-R019) (IFA: 1:200)
Rabbit anti-SARS-CoV-2 Spike S2 antibody (40590-T62) (WB: 1:2000)

The following antibody was purchased from BD Biosciences:
Mouse anti-EEA1 monoclonal antibody (Clone 14) (610456) (IFA: 1:100)

The following antibody was purchased from Abcam:
Rabbit anti-LY6E monoclonal antibody (Clone EPR26038-105)(ab300399) (WB: 1:1000)

The following antibody was purchased from GeneTex:
Mouse monoclonal antibody against SARS-CoV-2 Spike (Clone 1A9) (GTX632604) (IFA: 1:200)

The following antibodies were purchased from ThermoFisher:
Donkey anti-mouse IgG Alexa Fluoro-488 (A21202) (IFA: 1:500)
Donkey anti-rabbit IgG Alexa Fluoro-488 (A21206) (IFA: 1:500)
Donkey anti-mouse IgG Alexa Fluoro-568 (A10037) (IFA: 1:500)
Donkey anti-rabbit IgG Alexa Fluoro-568 (A10042) (IFA: 1:500)
Donkey anti-mouse IgG Alexa Fluoro-647 (A32787) (IFA: 1:500)
Donkey anti-rabbit IgG Alexa Fluoro-647 (A31573) (IFA: 1:500)
Donkey anti-Goat IgG horse-radish peroxidase-conjugated secondary antibody (PA1-28664) (WB: 1:5000)

The following antibodies were obtained from BEI Resources, NIAID, NIH:
Rabbit anti-SARS-CoV-2 spike monoclonal antibody (Clone number not available) (NR-53788) (IFA: 1:200)
Mouse anti-SARS-CoV-2 nucleocapsid monoclonal antibody (Clone 05) (NR-53792) (IFA: 1:200)
Rabbit anti-SARS-CoV-2 nucleocapsid monoclonal antibody (Clone 001) (NR-53791) (IFA: 1:200)

The following antibody was purchased from Jackson ImmunoResearch:
Goat anti-mouse Fab AF647 (115-607-003) (IFA: 1:200)

The following antibody was purchased from Biotium:
Goat anti-rabbit IgG CF660C (Cat# 20813) (IFA: 1:200)

Validation

Antibodies were validated by the manufacturers/providers as well as RRID database:
Rabbit anti-GAPDH monoclonal antibody (60004-1-Ig): https://www.ptglab.com/products/GAPDH-Antibody-60004-1-Ig.htm. RRID: AB-2107436.
Mouse anti-GFP tag monoclonal antibody (66002-1-Ig): https://www.ptglab.com/products/eGFP-Antibody-66002-1-Ig.htm. RRID: AB_11182611.
Rabbit anti-PLSCR1 polyclonal antibody (11582-1-AP): https://www.ptglab.com/products/PLSCR1-Antibody-11582-1-AP.htm. RRID: AB_2165659.
Mouse anti-Halo tag monoclonal antibody (28a8): https://www.ptglab.com/products/Halo-antibody-28A8.htm. RRID: AB_2827565.
Rabbit anti-TMEM41B polyclonal antibody (29270-1-AP): https://www.ptglab.com/products/TMEM41B-Antibody-29270-1-AP.htm. RRID:AB_2918264.
Rabbit anti-IFITM3 polyclonal antibody (11714-1-AP): https://www.ptglab.com/products/IFITM3-Antibody-11714-1-AP.htm. RRID:AB_2295684.
Rabbit anti-Na,K-ATPase polyclonal antibody (#3010S): https://www.cellsignal.com/products/primary-antibodies/na-k-atpase-antibody/3010. RRID:AB_2060983.
Rabbit anti-Flag tag monoclonal antibody (14793S): https://www.cellsignal.com/products/primary-antibodies/dykddddk-tag-d6w5b-rabbit-mab-binds-to-same-epitope-as-sigma-s-anti-flag-m2-antibody/14793. RRID:AB_2572291.

Goat anti-ACE2 polyclonal antibody (AF933): https://www.rndsystems.com/products/human-mouse-rat-hamster-ace-2-antibody_af933. RRID:AB_355722.

Mouse anti-PLSCR1 monoclonal antibody (MABS483): https://www.sigmaaldrich.com/US/en/product/mm/mabs483.
Mouse anti-dsRNA monoclonal antibody (MABE1134): https://www.sigmaaldrich.com/US/en/product/mm/mabe1134. RRID:AB_2819101.
Rabbit anti-SARS-CoV-2 nucleocapsid monoclonal antibody (40143-R019): https://www.sinobiological.com/antibodies/cov-nucleocapsid-40143-r019. RRID:AB_2827973.
Rabbit anti-SARS-CoV-2 Spike S2 antibody (40590-T62):https://www.sinobiological.com/antibodies/cov-spike-40590-t62
Mouse anti-EEA1 monoclonal antibody (610456): https://www.bdbiosciences.com/en-nz/products/reagents/microscopy-imaging-reagents/immunofluorescence-reagents/purified-mouse-anti-eea1.610456. RRID:AB_397829.
Rabbit anti-LY6E polyclonal antibody (ab300399): https://www.abcam.com/products/primary-antibodies/ly6esca-2-antibody-epr26038-105-ab300399.html
Mouse monoclonal antibody against SARS-CoV-2 Spike (GTX632604): https://www.genetex.com/Product/Detail/SARS-CoV-SARS-CoV-2-COVID-19-spike-antibody-1A9/GTX632604. RRID:AB_2864418.
Donkey anti-mouse IgG Alexa Fluoro-488 (A21202): https://www.thermofisher.com/antibody/product/Donkey-anti-Mouse-IgG-H-L-Highly-Cross-Adsorbed-Secondary-Antibody-Polyclonal/A-21202. RRID:AB_141607.
Donkey anti-rabbit IgG Alexa Fluoro-488 (A21206): https://www.thermofisher.com/antibody/product/Donkey-anti-Rabbit-IgG-H-L-Highly-Cross-Adsorbed-Secondary-Antibody-Polyclonal/A-21206. RRID:AB_2535792.
Donkey anti-mouse IgG Alexa Fluoro-568 (A10037): https://www.thermofisher.com/antibody/product/Donkey-anti-Mouse-IgG-H-L-Highly-Cross-Adsorbed-Secondary-Antibody-Polyclonal/A10037. RRID:AB_2534013.
Donkey anti-rabbit IgG Alexa Fluoro-568 (A10042): https://www.thermofisher.com/antibody/product/Donkey-anti-Rabbit-IgG-H-L-Highly-Cross-Adsorbed-Secondary-Antibody-Polyclonal/A10042. RRID:AB_2534017.
Donkey anti-mouse IgG Alexa Fluoro-647 (A32787): https://www.thermofisher.com/antibody/product/Donkey-anti-Mouse-IgG-H-L-Highly-Cross-Adsorbed-Secondary-Antibody-Polyclonal/A32787. RRID:AB_2762830.
Donkey anti-rabbit IgG Alexa Fluoro-647 (A31573): https://www.thermofisher.com/antibody/product/Donkey-anti-Rabbit-IgG-H-L-Highly-Cross-Adsorbed-Secondary-Antibody-Polyclonal/A-31573. RRID:AB_2536183.
Rabbit anti-SARS-CoV-2 spike monoclonal antibody (NR-53788): https://www.beiresources.org/Catalog/BEIMonoclonalAntibodies/NR-53788.aspx.
Mouse anti-SARS-CoV-2 nucleocapsid monoclonal antibody (NR-53792): https://www.beiresources.org/Catalog/BEIMonoclonalAntibodies/NR-53792.aspx.
Rabbit anti-SARS-CoV-2 nucleocapsid monoclonal antibody (NR-53791): https://www.beiresources.org/Catalog/BEIMonoclonalAntibodies/NR-53791.aspx.
Goat anti-mouse Fab AF647 (115-607-003): https://www.jacksonimmuno.com/catalog/products/115-607-003. RRID:AB_2338931.
Goat anti-rabbit IgG CF660C (Cat# 20813): https://biotium.com/product/goat-anti-rabbit-igg-hl-highly-cross-absorbed-cf-dye-storm/?attribute_pa_conjugation=cf660c.
Rabbit anti-TMEM16F polyclonal antibody (HPA038958): https://www.sigmaaldrich.com/US/en/product/sigma/hpa038958. RRID:AB_10672835
Sheep anti-mouse IgG horse-radish peroxidase-conjugated secondary antibody (GENXA931-1ML): https://www.sigmaaldrich.com/US/en/product/sigma/genxa9311ml. RRID:AB_772209.
Sheep anti-rabbit IgG horse-radish peroxidase-conjugated secondary antibody (GENA934-1ML): https://www.sigmaaldrich.com/US/en/product/sigma/gena9341ml. RRID:AB_772206.
Donkey anti-Goat IgG horse-radish peroxidase-conjugated secondary antibody (PA1-28664): https://www.thermofisher.com/antibody/product/Donkey-anti-Goat-IgG-H-L-Secondary-Antibody-Polyclonal/PA1-28664. RRID:AB_10990162.

Eukaryotic cell lines

Policy information about cell lines and Sex and Gender in Research

Cell line source(s)

Huh7.5 (kind gift from C. Wilen, commercially available in Apath LLC); A549-ACE2 (BEI Resources #NR-53821); Vero E6 (ATCC CRL-1586); HEK293T (ATCC CRL-3216); HeLa (ATCC CCL-2); Tonsil (UT-SCC-60A); HaCaT (kind gift from D. DiMaio, commercially available in AddexBio Technologies; #T0020001); LET1 (BEI Resources; NR-42941); hTEpiC (ScienCell #3220); Calu-3 (ATCC HTB-55).

Cell lines stably expressing human ACE2 (HeLa-ACE2, 293T-ACE2, Tonsil-ACE2 and HaCaT-ACE2) were generated from the aforementioned cell lines. For more information, please refer to the Methods part.

Authentication	Vero-E6, HEK293T, HeLa and Calu-3 cells were obtained from and pre-authenticated by ATCC and used at low passages. hTEpiC was obtained and pre-authenticated by ScienCell and used at low passages . A549-ACE2 and LET1 cells were obtained and pre-authenticated by BEI resource and used at low passages. CRISPR-Cas9 knockout cells constructed by us were validated by absence of protein expression by western blot. Cell morphology was used as authentication.
Mycoplasma contamination	All cell lines were tested routinely for mycoplasma contamination either by PCR analysis. If mycoplasma was detected, the cell line was discarded and any results acquired from the contaminated cell lines were reconfirmed in mycoplasma negative cells.
Commonly misidentified lines (See ICLAC register)	None.

Flow Cytometry

Plots

Confirm that:

☒ The axis labels state the marker and fluorochrome used (e.g. CD4-FITC).

☒ The axis scales are clearly visible. Include numbers along axes only for bottom left plot of group (a 'group' is an analysis of identical markers).

☒ All plots are contour plots with outliers or pseudocolor plots.

☒ A numerical value for number of cells or percentage (with statistics) is provided.

Methodology

Sample preparation	For CRISPR screening: Cells infected with SARS-CoV-2-mNeonGreen were fixed by 4% PFA for 30 min. Cells were washed 3 times by PBS. Cells were resuspended in FACS buffer (1xPBS, 1% FBS, 5mM EDTA) and filtered through a 40-µm cell strainer prior to FACS sorting using a BD FACSAria.<br><br>For PS externalization assay: Cells were digested by trypsin, spun down at 200 × g and washed twice with PBS. Cell pellets were resuspended in 100 µL of 1 × binding buffer (obtained from Thermo) at a density of 5×106 cells /mL and treated with DMSO or 10 µM ionomycin for 10 min. Cells were subsequently incubated with 5 µL Annexin V AF647 (Thermo) and DAPI for 20 min at room temperature followed by the addition of 400 µL 1× binding buffer. Cells were then analyzed using Beckman CytoFLEX S flow cytometer (APC filter).
Instrument	BD FACSAria was used to collect all data and perform cell sorting. Beckman CytoFLEX S flow cytometer was used to analyze flow cytometry data for PS externalization.
Software	BD FACSDiva (v.8) was used to collect flow cytometry data. FlowJo (v.10.2) was used to analyze data.
Cell population abundance	Populations sorted on mNeonGreen levels were estimated to be >95% pure based on analysis of post-sorting populations in pilot studies.
Gating strategy	The boundary for the mNeonGreen-High population was set based on maximal difference observed in wildtype cells between the interferon-γ treated and inteferon-γ non-treated samples.<br><br>For PS externalization assay, DAPI negative cells were selected. The cut-off of Annexin V AF647 positive cells was set based on the peak position in WT A549-ACE2 cells.

☒ Tick this box to confirm that a figure exemplifying the gating strategy is provided in the Supplementary Information.
