# Supplementary material for: PLSCR1 is a cell-autonomous defence factor against SARS-CoV-2 infection
Source: Nature. 2023 Jul 12;619(7971):819–27. doi: 10.1038/s41586-023-06322-y (PMC10371867; doi:10.1038/s41586-023-06322-y)
Supplement: Supplementary file 4 — sgRNA sequences used in this study. [file 41586_2023_6322_MOESM4_ESM.pdf]

Supplementary Table 1. sgRNA sequences used in this study.

**Target gene**	**sgRNA sequence (5’-3’)**
PLSCR1-1#	TATCCACCGACAGCATTCCA
PLSCR1-2#	TACTGAGGAGGATACCCAAC
PLSCR1-3#	CAACAGCTGCTACATCTTAG
STAT1	GAGGTCATGAAAACGGATGG
ACE2	AGATGTTACTGATGCAATGG
TMEM41B	TATGAAGGTTCCCAGAGATA
TMEM16F	AATAGTACTCACAAACTCCG
Negative control-1#	GACCGGAACGATCTCGCGTA
Negative control-2#	CGCTTCCGCGGCCCGTTCAA
Negative control-3#	ACGGAGGCTAAGCGTCGCAA
